# Comprehensive analysis and validation reveal DEPDC1 as a potential diagnostic biomarker associated with tumor immunity in non-small-cell lung cancer

**DOI:** 10.1371/journal.pone.0294227

**Published:** 2024-04-02

**Authors:** Meiwen Lv, Xuelian Li, Zhihua Yin, He Yang, Baosen Zhou

**Affiliations:** 1 Department of Clinical Epidemiology, The First Hospital of China Medical University, Heping District, Shenyang, China; 2 Department of Epidemiology, School of Public Health of China Medical University, Shenyang, China; Hokkaido University: Hokkaido Daigaku, JAPAN

## Abstract

Current evidence suggests that DEP domain containing 1 (DEPDC1) has an important effect on non-small-cell lung cancer (NSCLC). However, the diagnostic value and the regulatory function within NSCLC are largely unclear. This work utilized publicly available databases and in vitro experiments for exploring, DEPDC1 expression, clinical features, diagnostic significance and latent molecular mechanism within NSCLC. According to our results, DEPDC1 was remarkably upregulated in the tissues of NSCLC patients compared with non-carcinoma tissues, linked with gender, stage, T classification and N classification based on TCGA data and associated with smoking status and stage according to GEO datasets. Meanwhile, the summary receiver operating characteristic (sROC) curve analysis result showed that DEPDC1 had a high diagnostic value in NSCLC (AUC = 0.96, 95% CI: 0.94–0.98; diagnostic odds ratio = 99.08, 95%CI: 31.91–307.65; sensitivity = 0.89, 95%CI: 0.81–0.94; specificity = 0.92, 95%CI: 0.86–0.96; positive predictive value = 0.94, 95%CI: 0.89–0.98; negative predictive value = 0.78, 95%CI: 0.67–0.90; positive likelihood ratio = 11.77, 95%CI: 6.11–22.68; and negative likelihood ratio = 0.12, 95%CI: 0.06–0.22). Subsequently, quantitative real-time PCR (qRT-PCR) and western blotting indicated that DEPDC1 was high expressed in NSCLC cells. According to the in vitro MTS and apoptotic assays, downregulated DEPDC1 expression targeting P53 signaling pathway inhibited the proliferation of NSCLC cells while promoting apoptosis of NSCLC cells. Moreover, DEPDC1 was significantly correlated with immune cell infiltrating levels in NSCLC based on TCGA data, which were primarily associated with T cells CD4 memory activated, macrophages M1, B cells memory, mast cells resting, T cells regulatory, monocytes, and T cells CD4 memory resting. Compared with the group with high expression of DEPDC1, the group with low expression level had higher scores for immune checkpoint inhibitors (ICIs) treatment. GSEA confirmed that DEPDC1 was involved in gene expression and tumor-related signaling pathways. Finally, DEPDC1 and its associated immune-related genes were shown to be enriched in ‘receptor ligand activity’, ‘external side of plasma membrane’, ‘regulation of innate immune response’, and ‘Epstein-Barr virus infection’ pathways. The present study demonstrates that DEPDC1 may contribute to NSCLC tumorigenesis and can be applied as the biomarker for diagnosis and immunology.

## Introduction

Lung cancer represents a frequently seen cancer globally. It is estimated that 350 people are killed every day in the United States in 2022, therefore, lung cancer is the major factor resulting in cancer-associated mortality [1]. Pathologically, lung cancer can be classified as two major types, small cell lung cancer (SCLC) and NSCLC; NSCLC occupies approximately 85% of the lung cancer patients currently observed worldwide [2]. NSCLC comprises several frequently seen histological subtypes, like squamous cell carcinoma, large cell carcinoma and adenocarcinoma [3]. Despite major breakthroughs in the treatment of NSCLC in recent decades, clinicians still face difficulties in treating of advanced NSCLC, which is prone to relapse and metastasis, and the prognosis is very poor [4–6]. NSCLC remains one of the most aggressive and deadly cancer types.

DEPDC1 is a highly conserved protein among many species ranging from Caenorhabditis elegans to mammals that is located at 1p31.3 and plays a crucial role in regulating the proper mitotic progression [7, 8]. Moreover, DEPDC1 is primarily expressed in the testis and is hardly detected in other normal human tissues [9]. DEPDC1 is related to tumorigenesis and caner progression. Overwhelming evidence indicated the high expression of DEPDC1 in hepatocellular carcinoma samples relative to corresponding adjacent samples, which accelerates tumor cell growth but inhibits their apoptosis [10]. In addition, up-regulation of DEPDC1 in gastric cancer tissues has been associated with the advanced tumor differentiation and lymph node metastasis [11]. It has been increasingly suggested that DEPDC1 can be a key regulatory factor for different signaling pathways in tumors. Moreover, DEPDC1is up-regulated and plays a carcinogenic role in lung adenocarcinoma (LUAD) tissues, meanwhile, DEPDC1 up-regulates RAS expression in LUAD cells, thereby enhancing ERK1/2 activity and inhibiting autophagy via the RAS‐ERK1/2 signaling pathway [12]. DEPDC1 can act as an immunological biomarker in cancers. In this respect, it has been shown that DEPDC1 shows remarkable up-regulation within cancer samples in comparison with corresponding non-carcinoma samples, which is negatively related to dendritic cells during immune infiltration analysis in esophageal squamous cell carcinoma [13]. Immunotherapy has been applied in the treatment of cancers in recent years. Immune checkpoint inhibitors (ICIs) antibodies that target cytotoxic T lymphocyte antigen 4 (CTLA4) and PD1/PDL1 axis achieve remarkable successes in the treatment of NSCLC and are closely related to patient survival [14, 15]. At present, few studies have been reported on immunotherapy of DEPDC1 in NSCLC.

Herein, we assessed DEPDC1 expression levels within the tissues from NSCLC cases to illustrate its potential for application as a diagnostic and therapeutic biomarker and identified association between DEPDC1 expression and clinical features based on data from available open-access databases. Subsequently, we investigated the effects of DEPDC1 expression on biological behavior, targeting pathways, and related immune genes in vitro based on NSCLC cells. Finally, the obtained data were analyzed with an unabridged bioinformatics framework, including immune microenvironment, immune cell infiltration, immune checkpoint treatment, and signaling pathways with linked immune genes, to explore the possible immunological function of DEPDC1 in NSCLC.

## Materials and methods

### DEPDC1 expression analysis and data sources

This work collected RNA-sequencing data as well as related clinical data based on TCGA (https://portal.gdc.cancer.gov/). The sample inclusion criteria: completed DEPDC1 sequencing data in normal samples (108) and NSCLC samples (1037), and clinical data with 513 LUAD and 501 lung squamous cell carcinoma (LUSC), including age, gender, stage, TNM and race. This work “limma” package for detecting the difference of DEPDC1 expression between NSCLC and normal lung tissue samples.

Microarray about DEPDC1 in NSCLC was obtained from the GEO database (https://www.ncbi.nlm.nih.gov/geo/) with the following keywords: (malignant OR malignancy OR tumor OR tumour OR cancer OR carcinoma OR neoplasm OR neoplasms adenocarcinoma OR AC OR SCC OR NSCLC) AND (Lung OR pulmonary OR respiratory OR respiration OR bronchi OR bronchioles OR alveoli OR pneumocytes). The organism was screened by “Homo sapiens.” Criteria for selecting the subjects were as follows: (a) Each dataset involved lung cancerous and noncancerous samples; (b) The expression profiling data for DEPDC1 were available for both groups; (c) The normal and tumor groups included in tissue; (d) At least ten samples were included. Data from GEO were represented by mean ± standard deviation (SD), meanwhile, normalized and combined via R packages “limma” and “sva”.

Pan-cancer of DEPDC1 expression analysis was performed by TIMER2.0 (http://timer.cistrome.org/) which contained TCGA data.

### Data collection from the HPA database

The HPA database (https://www.proteinatlas.org/) contains the protein expression in normal cells, tissues, and cancers. This work conducted IHC for validating DEPDC1 expression within normal lung tissues and lung tumor tissues.

### Cells culture and treatment

BEAS-2B, NCI-H1299 and A549 were purchased from GeneChem (Shanghai, People’s Republic of China). Human normal lung epithelial cells (BEAS-2B) were cultured in RPMI-DMEM supplemented with 20% fetal calf serum (FBS). In addition, NCI-H1299 NSCLC cells and A549 LUAD cells were cultured within RPMI-1640 medium containing 10% FBS and transfected with a siRNA to knock down DEPDC1. Negative control siRNA (siRNA-NC) and siRNA targeting DEPDC1 were transfected into the NCI-H1299 and A549 cells for further experiments. The sequences were as follows:

**Table:** 

siRNA-DEPDC1: 5’- GGAAGAUGUUGAAGAAGUUTT – 3’
siRNA-NC: 5’ – UUCUCCGAACGUGUCACGUTT – 3’

### Quantitative real-time PCR and western blotting

This work extracted total RNA with RNAiso Plus (TAKARA 9108) and then transformed into cDNA by reverse transcription reaction (AG11711, Accurate Biology, China). Through adopting TAKARA RR820A kit, this work carried out qRT-PCR following specific protocols, with U6 being the internal controls. 2^-△△Ct^ approach was adopted for calculating transcription level of genes. The sequences of primers were as follows:

**Table:** 

DEPDC1 Forward: CTCGTAGAACTCCTAAAAGGCA
Reverse: TCAACATCTTCCTGGCTTAGTT
TNFSF12 Forward: CGCCAGATCGGGGAGTTTATAGTC Reverse: AGCACACCATCCACCAGCAAG
CD81 Forward: ACGAGACGCTTGACTGCTGTG Reverse: TTGAAGAGGTTGCTGATGATGTTGC
BIRC5 Forward: AAGGACCACCGCATCTCTACATTC Reverse: CTCGTTCTCAGTGGGGCAGTG
NRAS Forward: ACCAATACATGAGGACAGGCGAAG Reverse: ACTTGTTTCCCACTAGCACCATAGG

Total cellular proteins were obtained with RIPA lysis buffer containing the protease inhibitor (PMSF). Thereafter, protein separation was conducted on 10% SDS-PAGE before transfer on PVDF membranes. Later, membranes were blocked with skim milk, followed by overnight primary antibody incubation under 4°C, including polyclonal rabbit anti-DEPDC1 (bs-6525R; Bioss), polyclonal rabbit anti-P53 (Cat No. 10442-1-AP; Proteintech Group, Inc.), polyclonal rabbit anti-BAX (Cat No. 50599-2-Ig; Proteintech Group, Inc.) and anti-β-actin primary antibodies (Cat No. 66009-1-Ig; Proteintech Group, Inc.). Membranes were later washed thrice using 1×PBST, followed by another 1h incubation using the secondary antibody (Cat No. SA00001-1 and Cat No. SA00001-2; Proteintech Group, Inc.). Protein bands were detected by luminescent solution, recorded by the ECL system. This work utilized ImageJ software for quantifying gray values of protein bands.

### Cell proliferation and cell apoptosis assay

The present study inoculated NCI-H1299 cells in the 96-well plated, followed by siRNA transfection. At 0, 24, 48, and 72 h after transfection, 20ul MTS solution (Promega G3580, China) were added in per well, followed by 2 h incubation and measurement of absorbance at 490nm.

After siRNA transfection, apoptotic cell rate was quantified with Annexin V-APC/7-AAD (KGA1016, China) in line with specific instructions. EDTA-free pancreatin was added to collect cells, followed by washing trice with 1×PBS. Afterwards, 500ul binding buffer was added together with 5ul Annexin V-APC and 5ul 7-ADD to resuspend cells, followed by 5-15-min staining in dark. FCM (Flow cytometry, Germany) was conducted to detect apoptosis rate.

### Relation between DEPDC1 level and tumor immune microenvironment as well as immune infiltrating cells in NSCLC

StromalScore, ImmuneScore and ESTIMATE score were calculated by applying the ESTIMATE method approach of “estimate” and “limma” from R software, so as to predict diverse cell purity in tumor immune microenvironment using TCGA database. Meanwhile, the immune infiltrating cell levels in NSCLC were estimated using the CIBERSORT algorithm [16].

### The value of DEPDC1 in immunological therapy

Data related to PD1 and CTLA4 immunotherapy in NSCLC cohort were visualized from TCIA website (https://tcia.at/home). We utilized “limma” and ‘ggpubr’ packages of R soft for analyzing the differences between low-DEPDC1 and high-DEPDC1 groups in different immunotargets.

### GSEA

Transcriptomic data from TCGA were applied in GSEA. According to the expression level of DEPDC1 in NSCLC, all samples were classified as low- or high-expression group. GSEA software (version 4.1.0, Broad Institute) was employed for GSEA [17], with “c2.cp.kegg.v7.4.symbols.gmt” gene set from the MsigDB database being the reference. Pathways with FDR < 0.001 were considered significantly enriched.

### Immune-related genes of DEPDC1 in NSCLC

To predict the immune-related genes of DEPDC1, firstly, a total of 1793 immune-related genes were obtained according to the “Gene Lists” page of Immport database (https://www.immport.org/home). Then the correlation between DEPDC1 and abtained genes were calculated utilizing the Spearman method of R language based on TCGA data. At the criterion of *P* < 0.001 and |R| > 0.2, immune-related genes were considered as significantly correlated with DEPDC1 in NSCLC. Gene Ontology (GO) and Kyoto Encyclopedia of Genes and Genomes (KEGG) analysis were utilized by R software with cut-off threshold *p*-value = 0.05 and *q*-value = 1 to investigate pathway DEPDC1 and correlated gene enriched.

### Statistical analysis

IBM SPSS Statistics V23.0, Stata software (version 12.0), R software and GraphPad Prism Version 8.0.2 were employed for data analysis. Student’s test was adopted for statistical analysis. Chi-square test was adopted to analyze relationships between clinical characteristics and both groups. SPSS 23.0 was employed for constructing ROC curves. Stata12.0 and R software were used for meta-analysis that estimated the DEPDC1 expression and diagnostic value in NSCLC. The results were visualized in forest plots and sROC curve. Cochran’s Q (chi-square test) and the *I*^2^ test were applied to evaluate heterogeneity and ensure a suitable meta-analysis model was applied. Egger’s test was used to estimate the publication bias. Correlation analysis was completed by the method of Spearman (*P* < 0.001 and |R| > 0.2 as cut-off thresholds). *P* < 0.05 was stood for statistical significance.

## Results

### Confirmation of the expression, clinical and diagnostic effects of DEPDC1 in NSCLC, based on TCGA database

**Fig 1:**
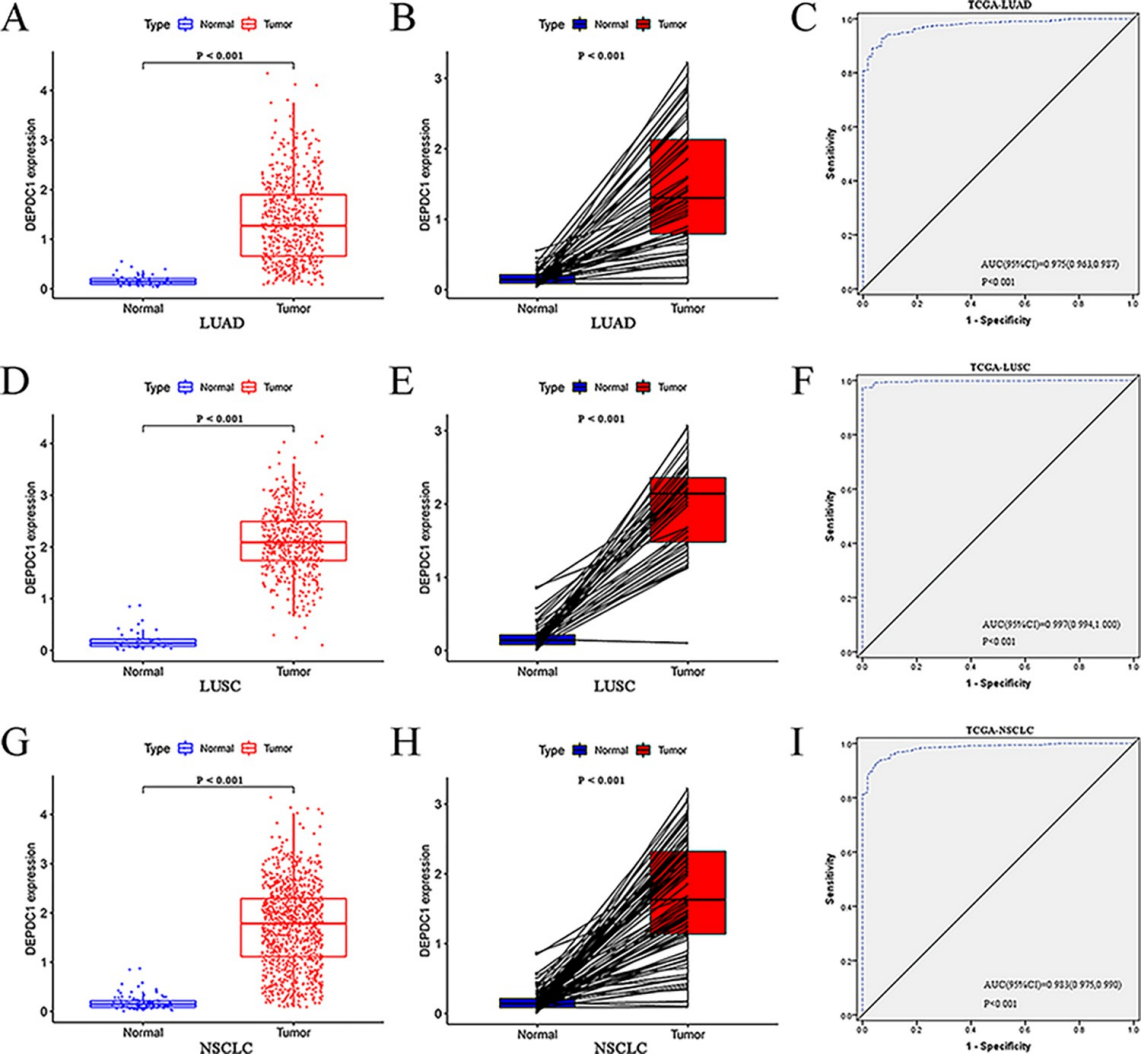
Expression and diagnostic value of DEPDC1 based on TCGA data. (A) Boxplots of DEPDC1 expression in LUAD. (B) Identification of DEPDC1 expression in pairs of LUAD samples. (C) The ROC curve of DEPDC1 in LUAD. (D) Boxplots of DEPDC1 expression in LUSC. (E) Identification of DEPDC1 expression in pairs of LUSC samples. (F) The ROC curve of DEPDC1 in LUSC. (G) Boxplots of DEPDC1 expression in NSCLC. (H) Identification of DEPDC1 expression in pairs of NSCLC samples. (I) The ROC curve of DEPDC1 in NSCLC.

**Fig 2:**
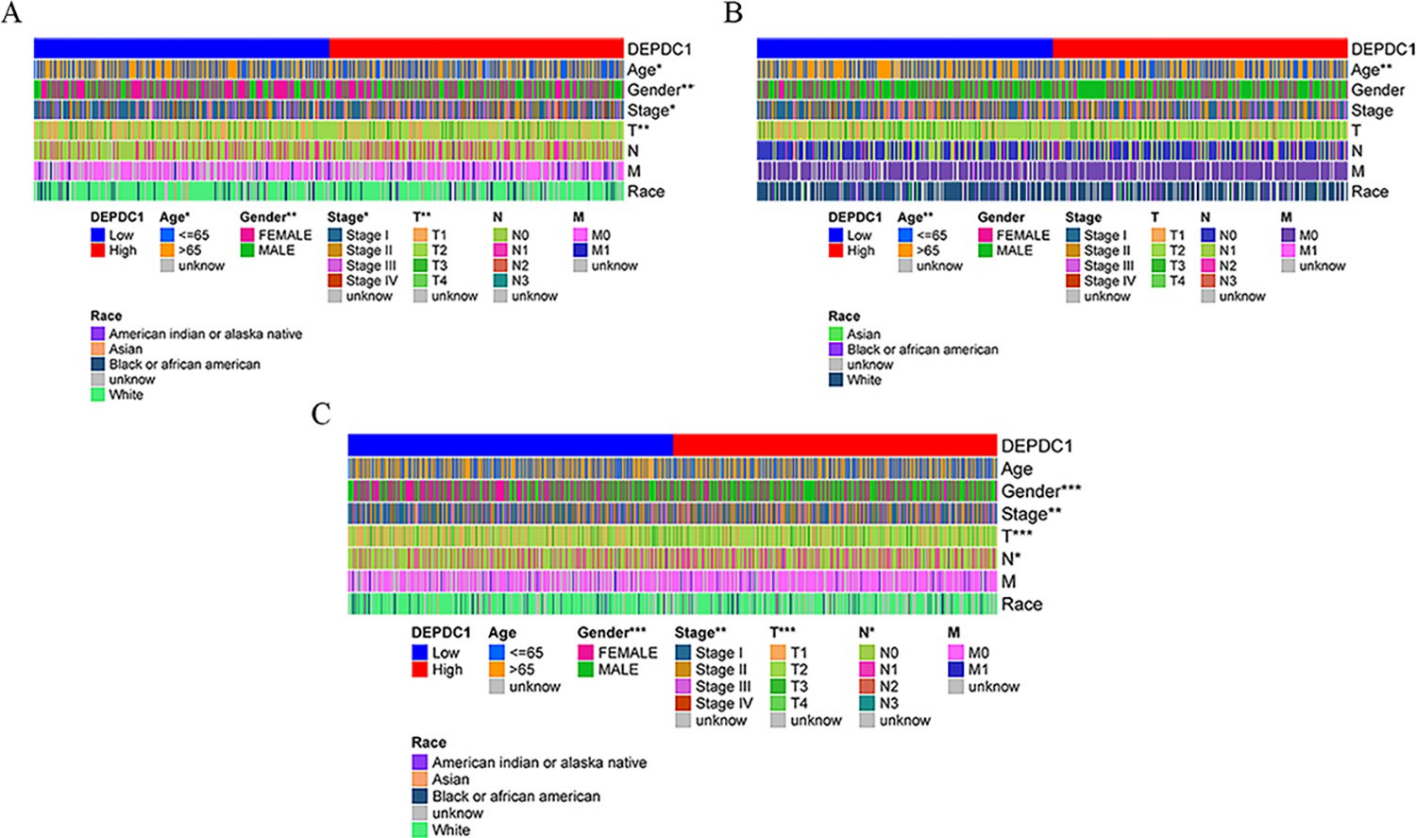
The heatmap of the clinical features of cases in the low- and high-DEPDC1 expression group in the TCGA cohort. (*P < 0.05, **P < 0.01, ***P < 0.001). (A) LUAD. (B) LUSC. (C) NSCLC.

The clinical characteristics of NSCLC patients were obtained from TCGA. DEPDC1 expression was significantly up-regulated in LUAD compared with non-carcinoma tissues (*P* < 0.001, Fig 1A and 1B). The AUC of upregulated DEPDC1 expression in LUAD was 0.975 (95%CI: 0.963, 0.987; *P* < 0.001, Fig 1C). The analyses of the LUSC groups alone revealed that DEPDC1 was upregulated in cancer tissues (*P* < 0.001, Fig 1D and 1E). The AUC of the high DEPDC1 level in the LUSC group was 0.997 (95%CI: 0.994, 1.000; *P* < 0.001, Fig 1F). The data on LUAD and LUSC, based on TCGA, were pooled for further validation. DEPDC1 expression was increased in NSCLC patients (*P* < 0.001, Fig 1G and 1H). The AUC of high DEPDC1 expression in NSCLC was 0.983 (95%CI: 0.975, 0.990; *P* < 0.001, Fig 1I). As for clinicopathological features of LUAD, age (*P* < 0.05), gender (*P* < 0.01), clinical stage (*P* < 0.05) and T classification (*P* < 0.01) were significantly associated with the expression of DEPDC1 (Fig 2A). And the abnormal DEPDC1 expression was significantly associated with age (*P* < 0.01) in LUSC patients (Fig 2B). As described in Fig 2C, a significant difference in DEPDC1 expression was found for gender (*P* < 0.001), clinical stage (*P* < 0.01), T classification (*P* < 0.001) and N classification (*P* < 0.05). Pan-cancer analysis using TIMER2.0 online tool found that DEPDC1 was highly expressed in NSCLC tissues, consistent with prior findings (S1 Fig).

### The expression, clinical pathology, and diagnostic value of DEPDC1 in NSCLC based on GEO datasets and meta-analysis

In total, 15 microarrays from GEO datasets satisfied the required criteria (GSE12236 GSE12428, GSE18842, GSE29250, GSE31210, GSE31446, GSE32863, GSE33532, GSE63459, GSE75037, GSE85716, GSE85841, GSE101929, GSE115002, and GSE134381). The features and analysis result of the contained GEO datasets were depicted in Table 1.

**Table 1:** Features of the eligible gene expression omnibus datasets.

Accession	GPL	Year	Country	Control			NSCLC			*P*-value	Source	Cancer type
				N	M	SD	N	M	SD			
GSE12236	GPL5188	2014	USA	20	2.120	0.068	20	3.370	1.114	**<0.001** ***	Tissue	LUAD
GSE12428	GPL1708	2012	Netherlands	28	-0.108	0.053	34	0.083	0.167	**<0.001** ***	Tissue	LUSC
GSE18842	GPL570	2019	Spain	45	3.464	0.120	46	5.572	0.930	**<0.001** ***	Tissue	NSCLC
GSE29250	GPL10558	2019	China	6	-18.375	9.435	6	-1.920	21.967	0.123	Tissue	NSCLC
GSE31210	GPL570	2019	Japan	20	25.191	13.468	226	119.811	135.339	**<0.001** ***	Tissue	LUAD
GSE31446	GPL9244	2012	USA	30	-1.075	0.320	34	-0.306	0.455	**<0.001** ***	Tissue	LUSC
GSE32863	GPL6884	2019	USA	58	6.792	0.054	58	6.923	0.137	**<0.001** ***	Tissue	LUAD
GSE33532	GPL570	2019	Germany	20	3.103	0.139	80	5.262	1.278	**<0.001** ***	Tissue	NSCLC
GSE63459	GPL6883	2017	USA	32	6.985	0.088	33	7.022	0.142	0.218	Tissue	LUAD
GSE75037	GPL6884	2019	USA	83	3.443	0.195	83	4.098	0.578	**<0.001** ***	Tissue	LUAD
GSE85716	GPL19612	2019	China	6	1.854	0.260	6	1.960	0.274	0.510	Tissue	LUAD
GSE85841	GPL20115	2018	China	8	45.240	23.985	8	26.780	18.269	0.105	Tissue	LUAD
GSE101929	GPL570	2021	USA	34	2.946	0.965	32	5.255	2.004	**<0.001** ***	Tissue	NSCLC
GSE115002	GPL13497	2021	China	52	3.423	0.550	52	4.983	1.231	**<0.001** ***	Tissue	LUAD
GSE134381	GPL11532	2019	United Kingdom	37	3.511	0.877	37	3.939	1.031	0.059	Tissue	NSCLC

LUAD Lung adenocarcinoma, LUSC lung squamous cell carcinoma, NSCLC non-small-cell lung cancer, M mean, SD standard deviation

*P < 0.05

**P < 0.01

***P < 0.001

**Fig 3:**
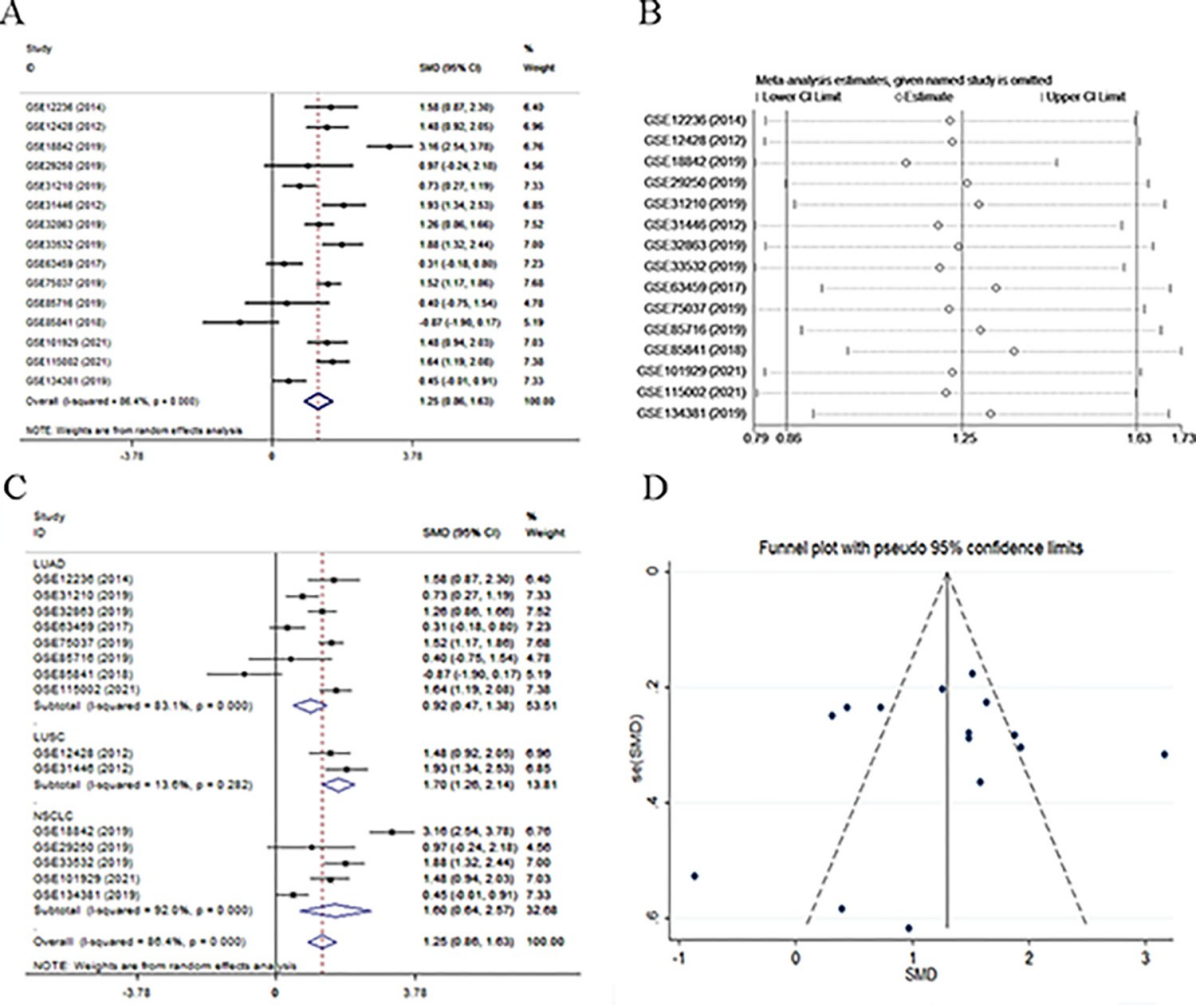
Continuous variable meta-analysis of GEO data. (A) Forest plot. (B) Sensitivity analysis. (C) Subgroup analysis based on cancer type. (D) Funnel plot.

Based on the 15 obtained microarrays, a meta-analysis was conducted (Fig 3A). In view of the high heterogeneity (*I*^2^ = 86.4%, *P* < 0.001), a random effects model was chosen. The meta-analysis of the 15 datasets that expression of DEPDC1 was remarkably increased in NSCLC groups than that in control groups (SMD = 1.25; 95% CI: 0.86, 1.63; *P* < 0.001). To detect the significant heterogeneity of an especial microarray, a sensitivity was performed (Fig 3B). After one individual study was eliminated, the combined effect of the remaining studies was compared with the previous studies, there was no particular one in the total studies. A subgroup analysis was conducted to further explore the source of heterogeneity on the basis of cancer type (Fig 3C). Significant heterogeneity was depicted in the LUAD subgroup (*I*^2^ = 83.1%, *P* < 0.001), LUSC subgroup (*I*^2^ = 13.6%, *P* = 0.282) and NSCLC subgroup (*I*^2^ = 92.0%, *P* < 0.001), which suggested that the types of cancer may be source of heterogeneity. Publication bias was estimated by Egger’s tests (*P* = 0.691) and shown by a funnel plot (Fig 3D). The results suggested no publication bias in the present meta-analysis.

After data combined based on GEO datasets, the clinical characteristics of 755 NSCLC samples and 479 normal control samples were shown in Table 2. The expression of DEPDC1 was significantly increased in LUAD, LUSC and NSCLC (*P* < 0.001). A significant difference in DEPDC1 was found for the smoking status (*P* < 0.001). Stage Ⅲ-Ⅳ NSCLC patients had a higher expression of DEPDC1 than stage Ⅰ-Ⅱ patients (*P* < 0.01).

**Table 2:** Relationship between the expression of DEPDC1 and clinicopathological features in NSCLC patients from GEO.

Clinicopathological feature	Type	N	M	SD	t/F	P-value
Tissue	Normal	479	3.836	0.560		
	LUAD	549	4.707	0.994	-17.582	**<0.001** ***
	LUSC	104	4.957	0.864	-12.658	**<0.001** ***
	NSCLC	755	4.799	0.982	-21.919	**<0.001** ***
Age	<65	363	4.807	1.050	1.541	0.124
	≥65	298	4.690	0.905		
Gender	Female	348	4.736	0.985	-0.520	0.603
	Male	319	4.776	0.998		
Tumor location	Central lung	18	4.948	0.877	-0.064	0.949
	Peripheral lung	50	4.962	0.790		
Smoking status	No	223	4.544	0.888	-4.400	**<0.001** ***
	Yes	293	4.913	1.013		
Stage	Ⅰ-Ⅱ	507	4.671	0.930	-3.215	**0.002** **
	Ⅲ-Ⅳ	60	5.158	1.129		
T	T1-T2	28	4.958	0.833	1.145	0.261
	T3-T4	6	4.551	0.504		
N	No	38	4.852	0.773	-1.332	0.189
	Yes	16	5.229	1.288		

LUAD Lung adenocarcinoma, LUSC lung squamous cell carcinoma, NSCLC non-small cell lung cancer, M mean, SD standard deviation

*P < 0.05

**P < 0.01

***P < 0.001.

**Fig 4:**
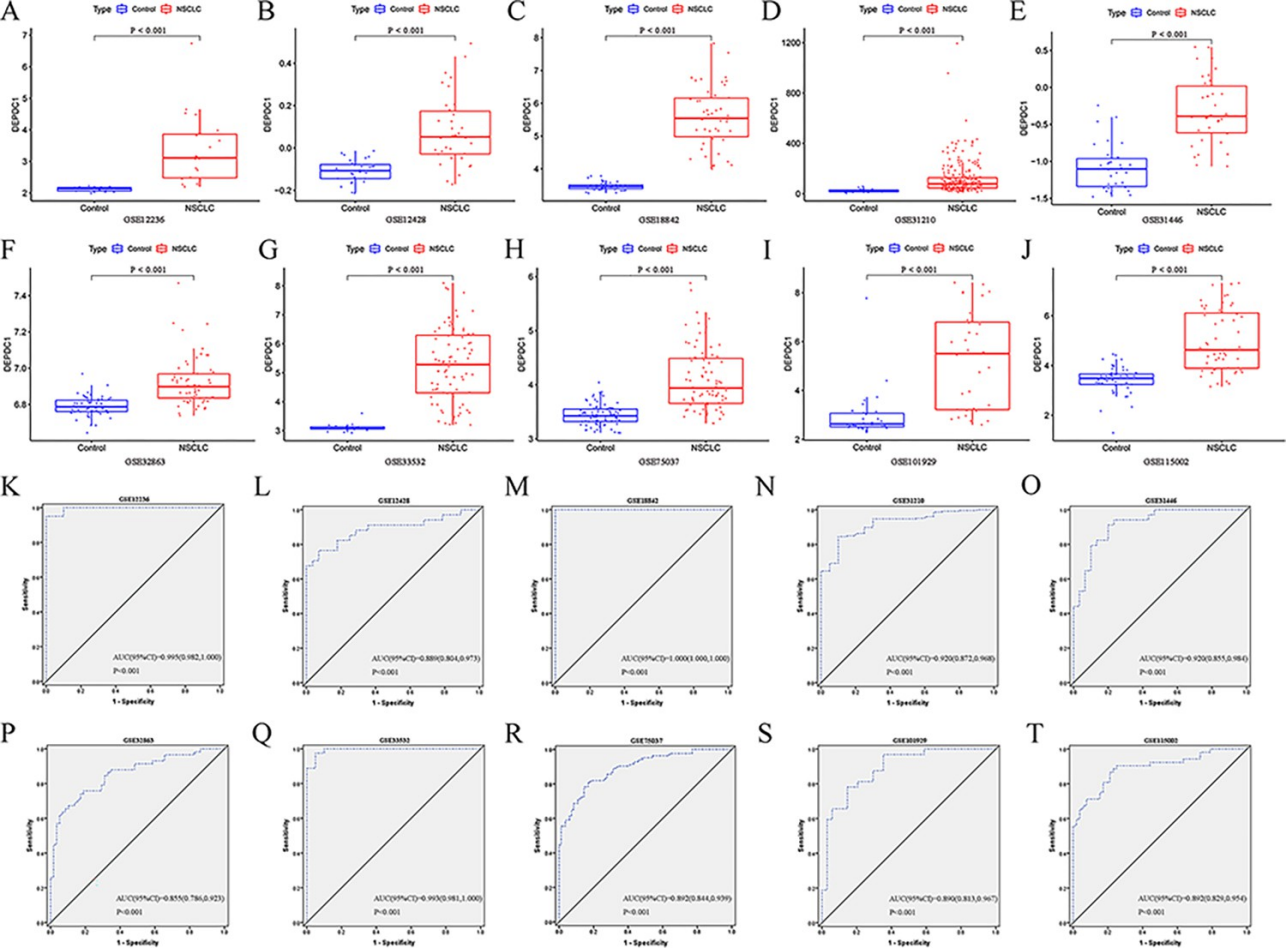
DEPDC1 expression in NSCLC and ROC curves of DEPDC1 based on GEO datasets. (A-J) DEPDC1 expression in NSCLC. (K-T) ROC curves of DEPDC1.

DEPDC1 (*P* < 0.001) showed high expression in NSCLC groups in 10 datasets, including GSE12236, GSE12428, GSE18842, GSE31210, GSE31446, GSE32863, GSE33532, GSE75037, GSE101929, and GSE115002 (Fig 4A–4J). And the p-value of the diagnostic power in the ROC curves based on the ten GEO datasets were all < 0.001 (Fig 4K–4T).

### Meta-analysis of the diagnostic value of DEPDC1 based on GEO and TCGA databases

**Fig 5:**
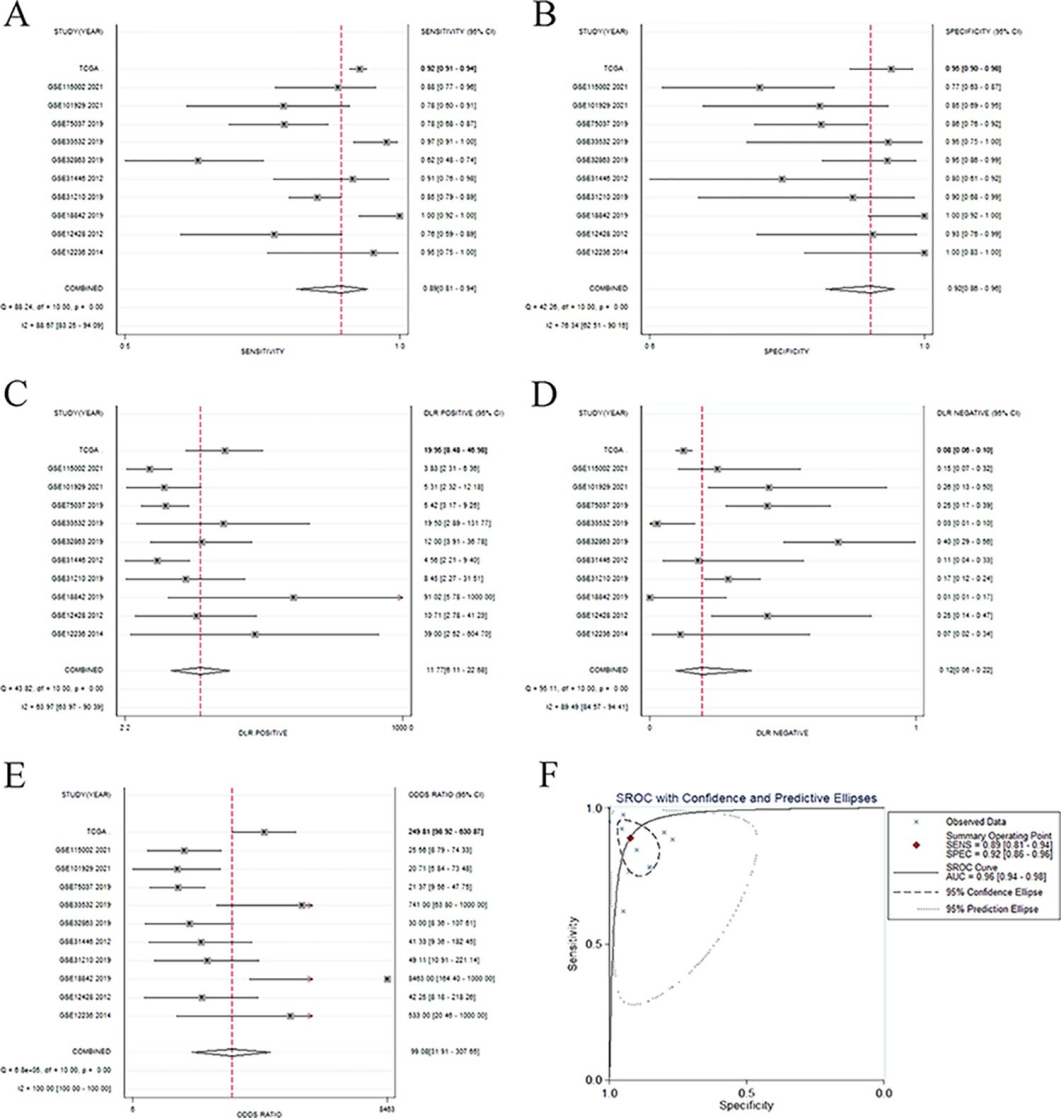
Forest plots and the sROC curve based on GEO and TCGA data. (A) Forest plot of sensitivity. (B) Forest plot of specificity. (C) Forest plot of positive likelihood ratio. (D) Forest plot of negative likelihood ratio. (E) Forest plot of odds ratio. (F) The sROC curve.

A total of 10 microarrays from GEO datasets and TCGA mRNA-sequencing data were included in the diagnostic meta-analysis. Forest plots results of relevant data on sensitivity, 0.89 (95%CI: 0.81–0.94); specificity, 0.92 (95%CI: 0.86–0.96); positive likelihood ratio, 11.77 (95%CI: 6.11–22.68); negative likelihood ratio, 0.12 (95%CI: 0.06–0.22) and diagnostic odds ratio, 99.08 (95%CI: 31.91–307.65) of DEPDC1 in diagnosing NSCLC were displayed in Fig 5A–5E. Meanwhile, the sROC curve yielded an AUC value of 0.96 (95%CI: 0.94–0.98, Fig 5F). Moreover, the pooled positive predictive value (PPV) and negative predictive value (NPV) calculated by R language with random effects model were 0.94 (95%CI: 0.89–0.98) and 0.78 (95%CI: 0.67–0.90), respectively.

### Protein expression verified through IHC

**Fig 6:**
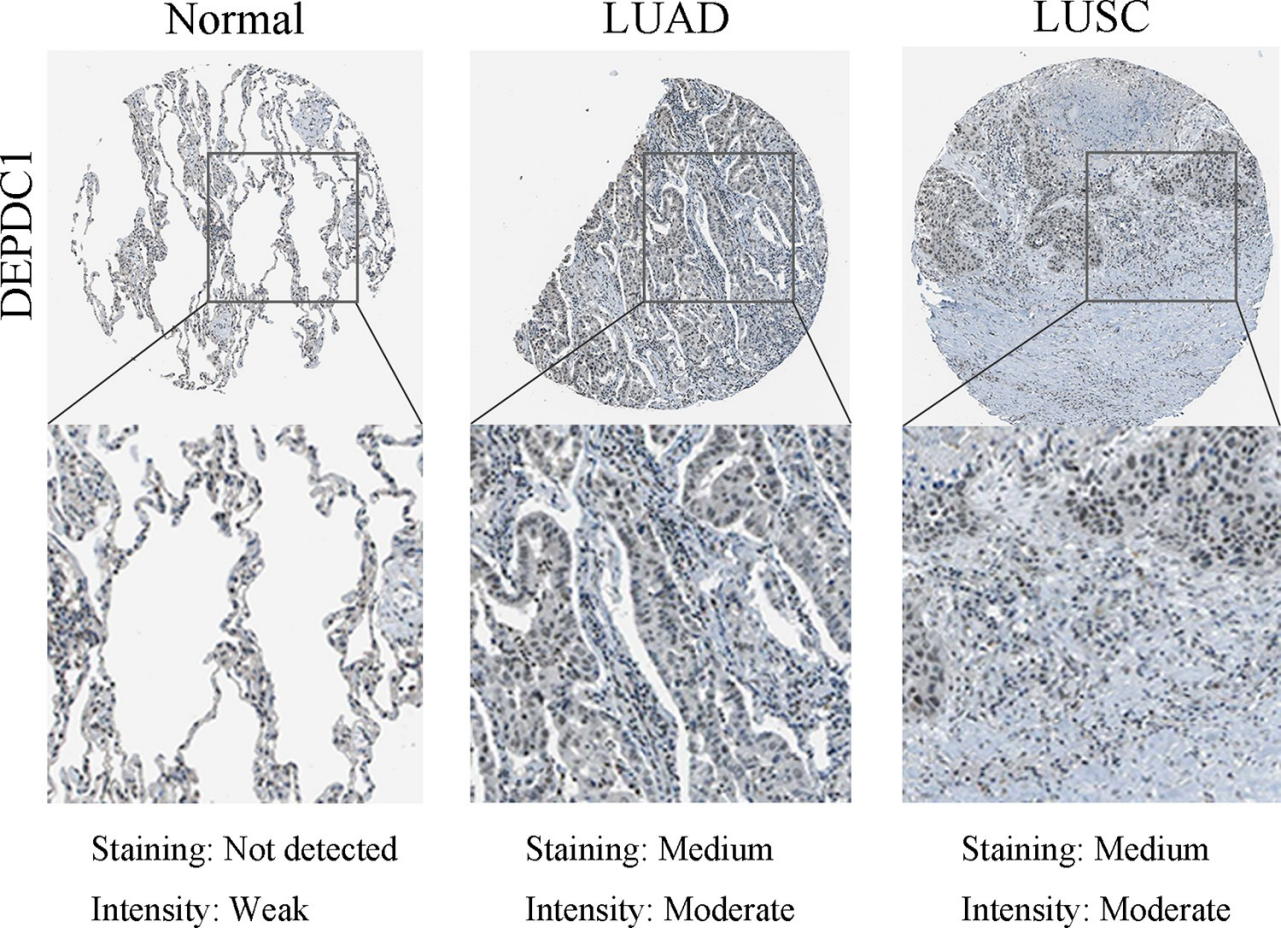
Immunohistochemical analysis of DEPDC1 in the HPA-derived normal, LUSC and LUAD samples.

According to Fig 6, up-regulated DEPDC1 expression was detected in tumor tissues in comparison with normal tissues.

### Decreased DEPDC1 expression suppressed NSCLC cells growth and enhanced their apoptosis

**Fig 7:**
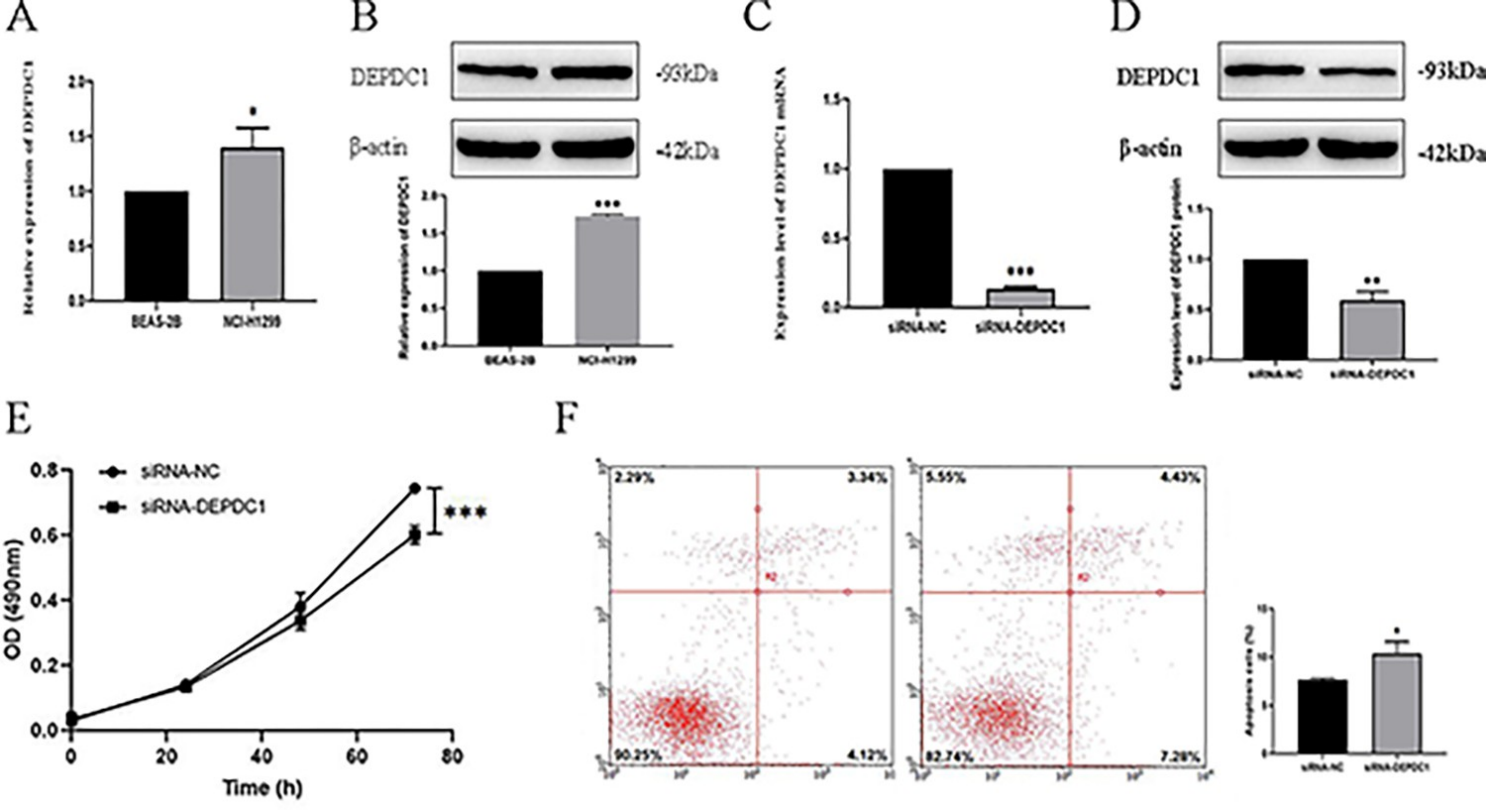
DEPDC1 inhibits NSCLC cell proliferation and enhances their apoptosis (*P < 0.05, **P < 0.01, ***P < 0.001). (A) DEPDC1 mRNA expression within BEAS-2B and NCI-H1299 cells. (B) DEPDC1 protein expression within BEAS-2B and NCI-H1299 cells. (C) DEPDC1 mRNA expression within NCI-H1299 cells after siRNA-NC and siRNA-DEPDC1 transfection. (D) DEPDC1 protein expression within NCI-H1299 cells after siRNA-NC and siRNA-DEPDC1 transfection. (E) MTS assay conducted to analyze NCI-H1299 cell proliferation at 0/24/48/72h after siRNA-NC and siRNA-DEPDC1 transfection. (F) NCI-H1299 cell apoptosis after siRNA-NC and siRNA-DEPDC1 transfection.

First, we found that DEPDC1 mRNA (Fig 7A: *P* < 0.05) and protein (Fig 7B: *P* < 0.001) levels were up-regulated in NCI-H1299 cells compared with BEAS-2B cells. Afterwards, for exploring the effect of DEPDC1 on cancer cells, DEPDC1 was silenced in NCI-H1299 cells. As a result, DEPDC1 mRNA (Fig 7C: *P* < 0.001) and protein (Fig 7D: *P* < 0.01) expression remarkably decreased in siRNA-DEPDC1 groups relative to the siRNA-NC groups. According to MTS analysis, DEPDC1 down-regulation could suppressed NCI-H1299 cell proliferation relative to control group (Fig 7E: *P* < 0.001). On the contrary, for NCI-H1299 cells showing DEPDC1 down-regulation, the apoptosis rate remarkably increased relative to control group (Fig 7F: *P* < 0.05).

### Immunological function of DEPDC1

**Fig 8:**
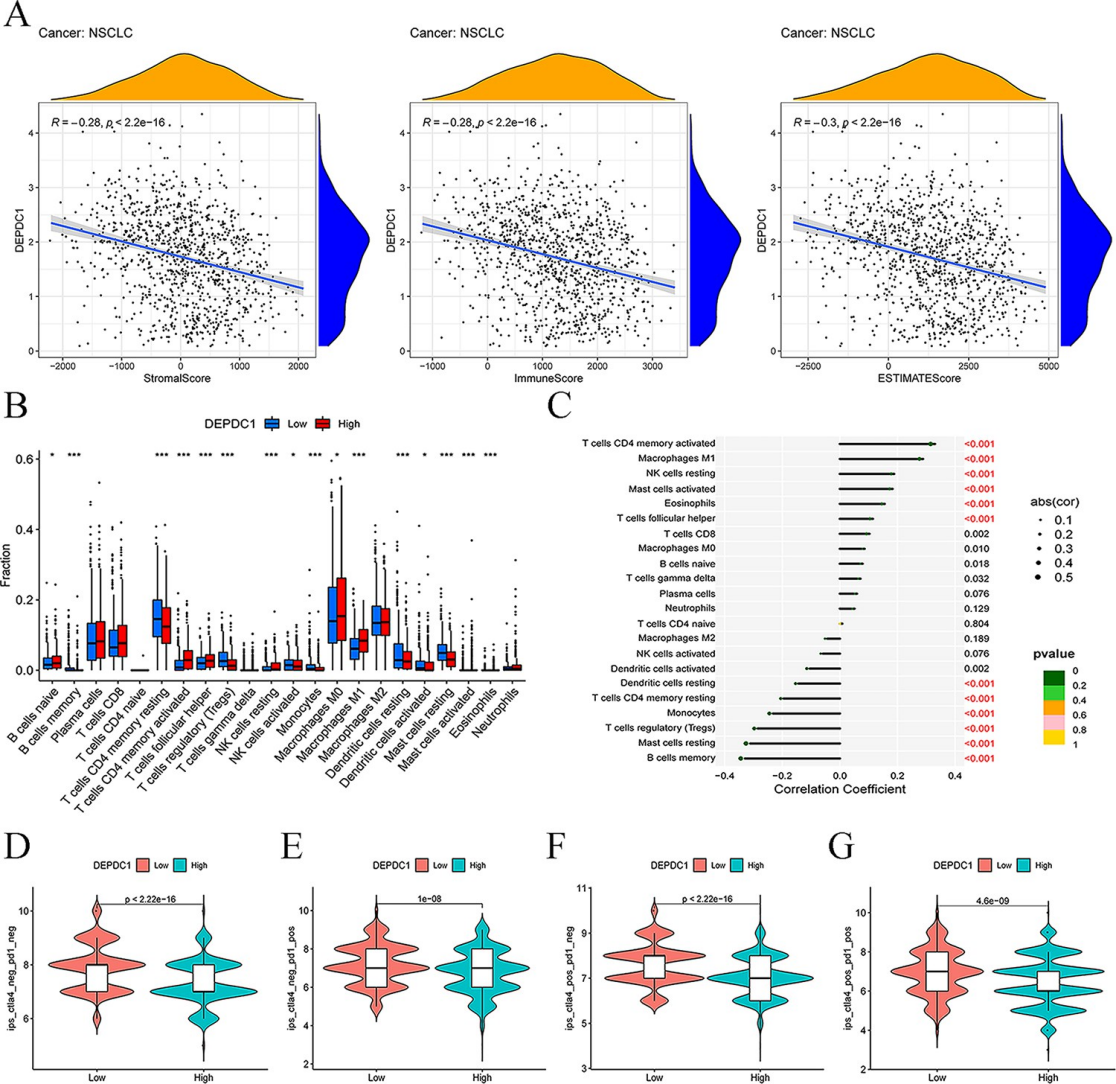
Relations between DEPDC1 level and tumor immune microenvironment, immune infiltrating cells and immune checkpoint inhibitors based on TCGA database (*P < 0.05, **P < 0.01, ***P < 0.001). (A) Scatter diagrams showing the correlation between DEPDC1 expression and tumor immune microenvironment. (B) Boxplots displaying the immune infiltrating cells between the low- and high-DEPDC1 expression groups in NSCLC. (C) Lollipop plot showing the relation of DEPDC1 level with immune infiltrating cells within NSCLC. (D-G) Relation of DEPDC1 level with immune checkpoint inhibitors.

This work also explored the relations among tumor immune microenvironment, immune cell infiltration, immunotherapeutic targets and DEPDC1 level. Typically, the relations of DEPDC1 expression with diverse infiltrating cell types within the NSCLC microenvironment were analyzed by StromalScore, ImmuneScore and ESTIMATEScore using the ESTIMATE algorithm. As showed in Fig 8A, DEPDC1 expression showed significant relation to StromalScore, ImmuneScore and ESTIMATEScore (*P* < 0.001) in NSCLC. Different infiltrating immune cells could be detected within tumor immune microenvironment. DEPDC1 was positively correlated with T cells CD4 memory activated, macrophages M1, while, negatively correlated with B cells memory, mast cells resting, T cells regulatory, monocytes, and T cells CD4 memory resting (Fig 8B and 8C). As for immunotherapy, we detected treatment scores for immune checkpoint inhibitors, and discovered statistically significant difference between low-DEPDC1 and high-DEPDC1 groups with no CTLA4 or PD1 treatment (Fig 8D, *P* < 0.001). In addition, PD1 or CTLA4 monotherapy exhibited increased immune scores in low-DEPDC1 group relative to high-DEPDC1 group, with significant difference (Fig 8E and 8F, *P* < 0.001). After PD1 was used in combination with CTLA4, the low-DEPDC1 group had an increased immune score (Fig 8G, *P* < 0.001).

### GSEA of gene DEPDC1

**Fig 9:**
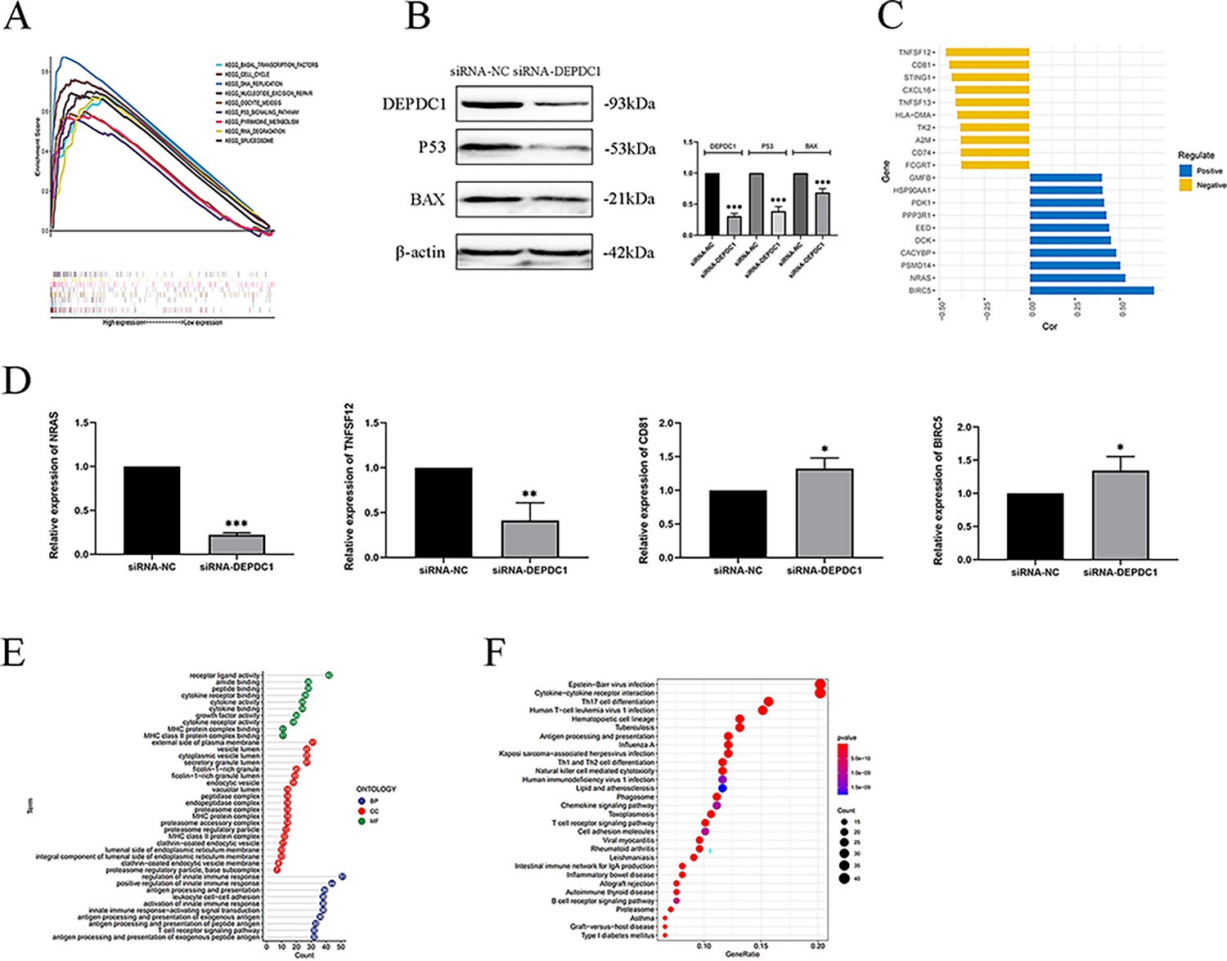
Analysis of DEPDC1 signaling pathway and immune-related genes (*P < 0.05, **P < 0.01, ***P < 0.001). (A) Gene enrichment plot of multiple pathways in GSEA. (B) Protein levels of DEPDC1, P53 and BAX in response to the treatment of siRNA-NC and siRNA-DEPDC1. (C) Deviation plot of the top 20 immune-related genes significantly associated with DEPDC1. (D) The expression levels of NRAS, TNFSF12, CD81 and BIRC5 mRNAs after the treatment of siRNA-NC and siRNA-DEPDC1. (E) GO analysis of DEPDC1 and immune-related genes. (F) KEGG pathways items of DEPDC1 and immune-related genes.

GSEA performed with TCGA showed that NSCLC samples with high expression DEPDC1 were enriched in ‘BASAL TRANSCRIPTION FACTORS’, ‘CELL CYCLE’, ‘DNA REPLICATION’, ‘NUCLEOTIDE EXCISION REPAIR’, ‘OOCYTE MEIOSIS’, ‘P53 SIGNALING PATHWAY’, ‘PYRIMIDINE METABOLISM’, ‘RNA DEGRADATION’ and ‘SPLICEOSOME’ pathways (Fig 9A). NCI-H1299 is a congenital P53-deficient cell line, therefore, the A549 cell line was used to detect the relationship between DEPDC1 expression and the associated P53 signaling pathway. The P53 and BAX protein levels decreased in A549 cells, after siRNA treatment, DEPDC1 protein expression was silenced within A549 cells (Fig 9B). These results suggest that DEPDC1 regulated the P53 signaling pathway.

### DEPDC1 immune-related genes as well as relevant pathway within NSCLC

Based on ImmPort database, 245 immune-related genes that correlated with DEPDC1 were identified and the top 20 were showed in Fig 9C. We checked the expression levels of top four correlated with DEPDC1 after siRNA treatment in NCI-H1299 cells (Fig 9D). The results of qRT-PCR showed that mRNA levels of NRAS and TNFSF12 were decreased after knocking down DEPDC1 expression. On the contrary, the expression levels of BIRC5 and CD81 mRNA were increased after knocking down DEPDC1 expression. Influenced by DEPDC1, mRNA levels of CD81 and NRAS showed the same trend as predicted based on TCGA data, while TNFSF12 and BIRC5 were contrary to the predicted trend.

GO analysis conducted on the eligible 245 genes and DEPDC1 were enriched in biological process (BP), including ‘receptor ligand activity’, ‘amide binding’ and ‘peptide binding’, ‘external side of plasma membrane’, ‘vesicle lumen’, and ‘cytoplasmic vesicle lumen’ in cellular component (CC) category, additionally included ‘regulation of innate immune response’, ‘positive regulation of innate immune response’, and ‘antigen processing and presentation’ in molecular function (MF) category (Fig 9E). During KEGG pathway analysis, ‘Epstein-Barr virus infection’, ‘Cytokine-cytokine receptor interaction’, and ‘Th17 cell differentiation’ were significantly enriched (Fig 9F).

## Discussion

Although DEPDC1 expression in NSCLC has been reported, few studies have documented the correlation of the clinical features of NSCLC and DEPDC1, diagnostic value, and the latent mechanism of DEPDC1 in NSCLC. In the present study, meta-analysis, bioinformatics analysis and in vitro were employed to investigate the role of DEPDC1 in NSCLC.

DEPDC1 shows up up-regulation within multiple cancer, like gastric cancer [11], hepatocellular carcinoma [18], triple-negative breast cancer [19], prostate cancer [20] and NSCLC [21]. According to our results, DEPDC1 up-regulation found within NSCLC was verified by publicly available databases and NSCLC cells. Thus, the results suggested that DEPDC1 might function as a promoter in NSCLC. Based on GEO database analysis, DEPDC1 was associated with smoking status and tumor stage. Moreover, TCGA data analysis indicated that DEPDC1 expression level was related to gender, clinical stage, T classification and N classification in NSCLC. Besides, AUC values from GEO and TCGA data were greater than 0.8 (*P* < 0.001); thus, GEO- and TCGA-based diagnosis analysis and meta-analysis suggested that DEPDC1 yielded a relatively high diagnostic efficiency in differentiating NSCLC patients from controls.

It has been reported that, DEPDC1 is a key gene in the transformation of hepatitis B into hepatocellular carcinoma [22]. Chronic inflammation can lead to the continuous activation of immune cells, interfere with the invasion of tumor immune cells and inhibit the anti-tumor immune response of tumor [23]. An increasing body of evidence suggests that immune cells in the tumor immune microenvironment have important effects on tumorigenesis and tumor development [24]. In clear cell renal cell carcinoma, resting mast cells are positively correlated with MUC20 level in the tumor immune microenvironment, while MUC20 expression is negatively linked with activated CD4+ memory T cells [25]. In LUAD, it has been reported that p53 mutation can affect the abundance of macrophages M1 in tumor-infiltrating lymphocytes [26], and memory B cells are significantly associated with NCAPH expression [27]. Interestingly, regulatory T cells are related to CD3E in bladder cancer [28]. Monocytes are regulated by CCR2 in the tumor immune microenvironment of sarcoma [29]. PLXDC2 expression is related to T cell CD4 memory resting proportion within gastric cancer [30]. In NSCLC, DEPDC1 is associated with T cells CD4 memory activated, macrophages M1, B cells memory, mast cells resting, T cells regulatory, monocytes, and T cells CD4 memory resting.

In this paper, the treatment scores of ICIs were significantly associated DEPDC1 expression. Immunotherapy targeting ICIs have achieved phased success in clinical tumor therapy, however, there are still some patients with poor drug resistance treatment [31, 32]. Reversing T-cell exhaustion caused by conditions such as chronic infection and cancer is a promising immunotherapy for cancer, which exerts a distinct anticancer activity, as detected in PD1 immunotherapy, moreover, immune checkpoint inhibitors plus T-cell activators have been suggested to generate the prominent anticancer immunity [33, 34]. PD1 combined with reversing T-cell exhaustion is of great significance for the immunotherapy of patients with NSCLC.

Few recent studies have documented the biological function of DEPDC1 within NSCLC. According to our results, GSEA revealed the significant enrichment of high-DEPDC1-expression group in ‘BASAL TRANSCRIPTION FACTORS’, ‘CELL CYCLE’, ‘DNA REPLICATION’, ‘PYRIMIDINE METABOLISM’, ‘NUCLEOTIDE EXCISION REPAIR’, ‘OOCYTE MEIOSIS’, ‘P53 SIGNALING PATHWAY’, ‘RNA DEGRADATION’ and ‘SPLICEOSOME’. Basal transcription factors have been associated with transcription mediated by RNA polymerase II, which transcribes all protein-coding genes in eukaryotic genomes [35, 36]. In gastric cancer, the cell cycle signaling pathway can be activated by overexpressing CDK1, which ultimately affects the development of gastric cancer cells and then affects cancer progression [37]. DNA replication plays a central role in genome health; the errors during the DNA replication process leads to various diseases and even tumors [38]. Pyrimidine metabolism is a critical metabolic pathway associated with DNA replication in tumor cells, and it plays an important role in tumor development [39]. With regard to DNA lesions with different structures, cells can reverse the genome into the original form through using the nucleotide excision repair (NER) pathway with high conservation degree; moreover, it has been reported that the NER pathway was associated with cisplatin resistance in NSCLC cell lines [40]. The oocyte meiosis pathway is enriched by different cancer-related genes [41, 42]. The p53 signaling pathway has been extensively studied and proved to be associated with proliferation migration apoptosis of various cancer cells, suggesting it is a potential immunotherapy target [43, 44]. It has been established that the RNA degradation system ensures the normal expression of genes; an error in the RNA degradation process can interfere with gene transcription and translation of various biological processes and ultimately lead to the occurrence of different diseases [45]. The spliceosome pathway is related to regulating gene levels and has been proved to related to various tumors, and its abnormal changes may affect tumor immune response, prognosis, immunotherapy and targeted drug therapy [46]. In this paper, DEPDC1 was targeting P53 signaling pathway in NSCLC cells. These findings revealed that DEPDC1 is mainly involved in cancer-related signaling pathways and DNA replication, repair, transcription and translation signaling pathways, suggesting that DEPDC1 might be related to the molecular mechanism of genesis and development of NSCLC, representing a potential therapeutic target.

To further explore the underlying molecular mechanisms, GO and KEGG analyses were performed to reveal the enrichment of DEPDC1 and immune-related genes. The GO terms were enriched in ‘receptor ligand activity’ for BP, ‘external side of plasma membrane’ for CC and ‘regulation of innate immune response’ for MF, revealing that the genes might have important effects on the targeted regulation of tumorigenesis, progression and immunotherapy [47, 48]. Epstein-Barr virus (EBV) is tightly associated with cancer genesis, which participates in the oncogenic and immune signaling pathways of tumors and may be a potential target for immunotherapy and drug therapy [49–51]. As for KEGG analysis, ‘Epstein-Barr virus infection’ pathway was the most significantly enriched. Overall, these findings may provide new directions to understand tumor occurrence and therapeutic mechanisms.

However, certain limitations should be noted in this work. First, data from TCGA only contained tissue sample data. Besides, in vivo experiments of DEPDC1 are lacking. Moreover, the pathways were based on data from different datasets rather than experimental verification. Therefore, further experiments are warranted to substantiate our findings.

## Conclusion

In summary, DEPDC1, which was of high diagnostic value, was up-regulated in NSCLC tissues, and was associated with gender, stage, T classification, N classification and smoking status according to meta-analysis based on TCGA and GEO databases. Through the in vitro experiments, the DEPDC1 mRNA and protein expression increased in NSCLC cells; besides, the down-regulated DEPDC1 expression could inactivate the P53 signaling pathway, which thereby inhibited cell growth and promoted their apoptosis. Moreover, DEPDC1 was significantly correlated with immune cell infiltrating levels and immune-related genes. The results of bioinformatics analysis and in vitro experiments suggested that DEPDC1 may be a key diagnostic and immunological marker, which provided a new method for investigating the pathogenesis, development and immunotherapy response of NSCLC.

## Supporting information

S1 Fig The expression levels of DEPDC1 in different cancers based on TIMER2.0 database. (*P < 0.05, **P < 0.01, ***P < 0.001).
(TIF)

S1 Raw images The original uncropped and unadjusted images underlying all blot results. (PDF)
