# Supplementary figures and images for: Comprehensive analysis and validation reveal DEPDC1 as a potential diagnostic biomarker associated with tumor immunity in non-small-cell lung cancer
Source: PLoS One. 2024 Apr 2;19(4):e0294227. doi: 10.1371/journal.pone.0294227 (PMC10986975; doi:10.1371/journal.pone.0294227)

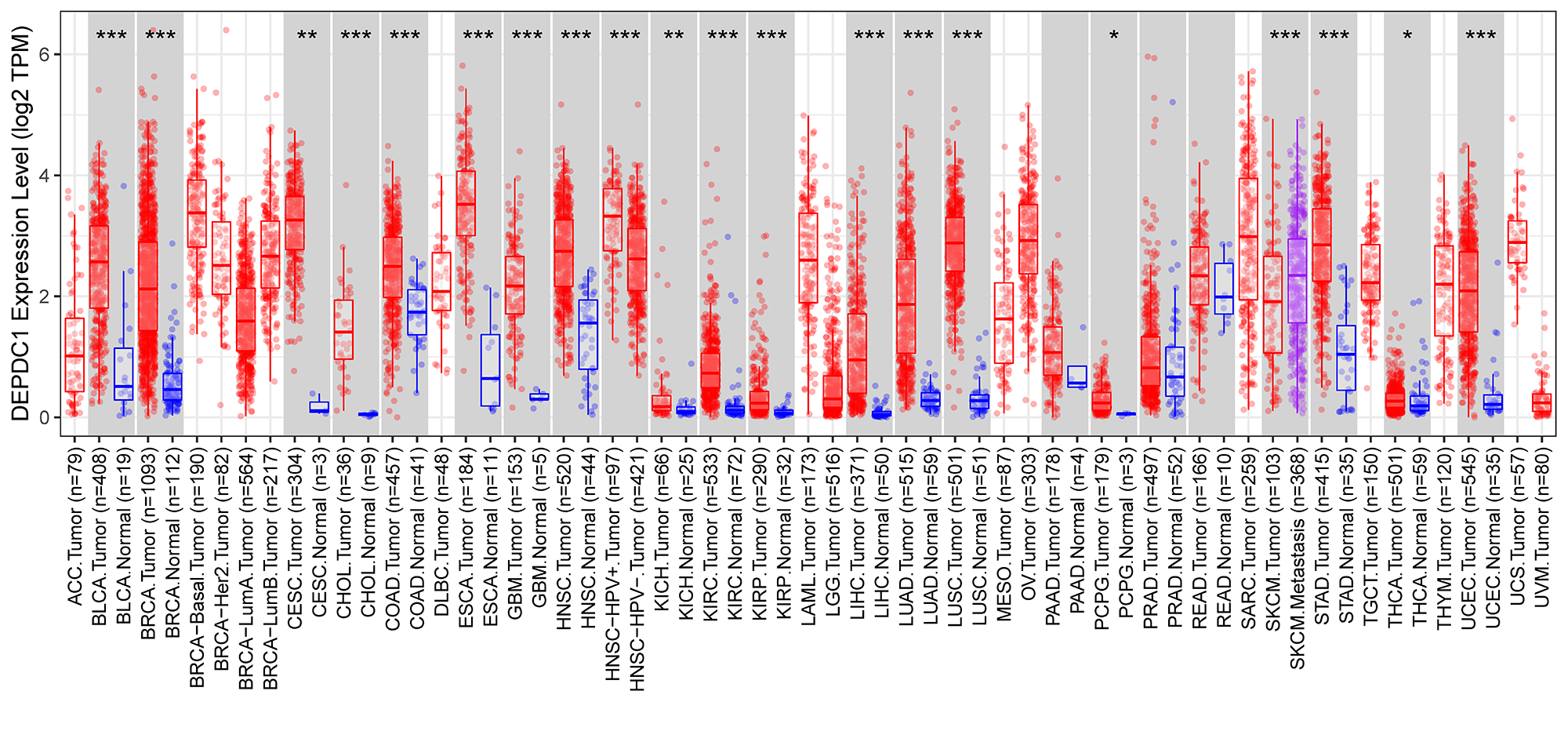

Supplement: S1 Fig — (*P < 0.05, **P < 0.01, ***P < 0.001). (TIF) [file pone.0294227.s001.tif]

**Fig 7B and Fig 7D**

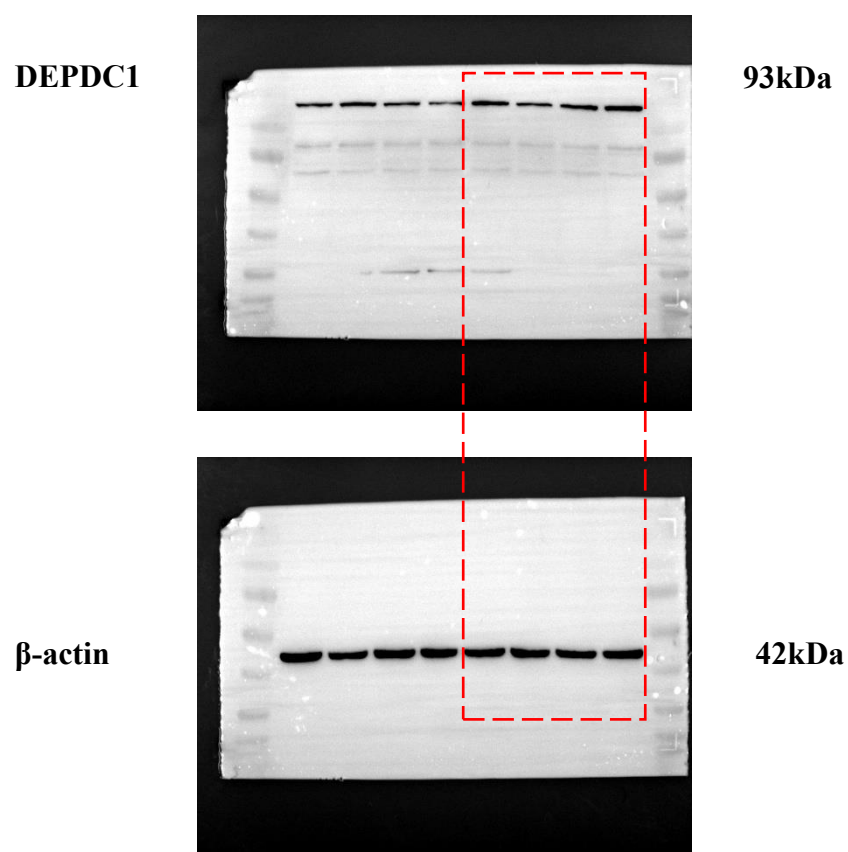

**Fig 9B**

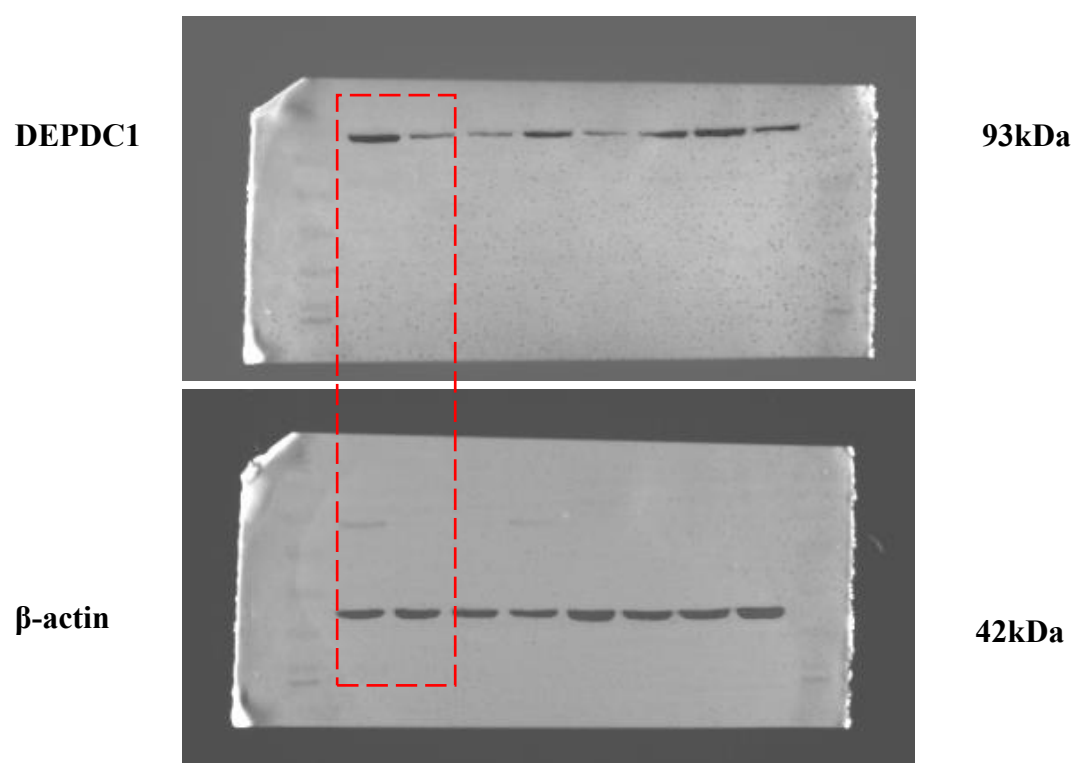

**P53**

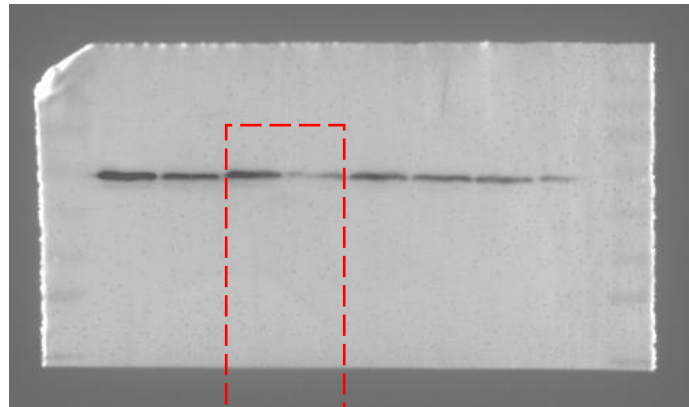

**53kDa**

**$\beta$ -actin**

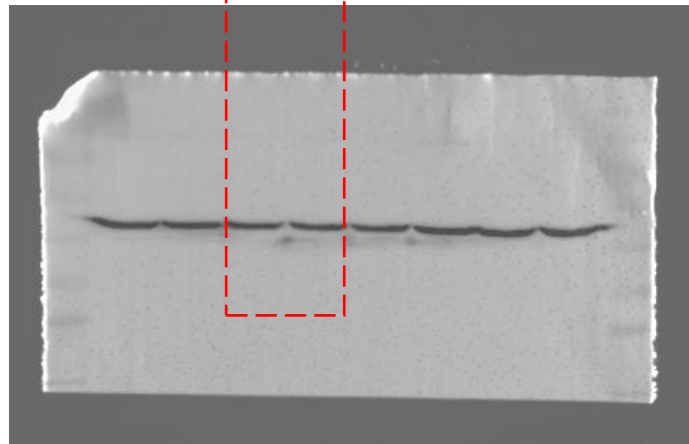

**42kDa**

**BAX**

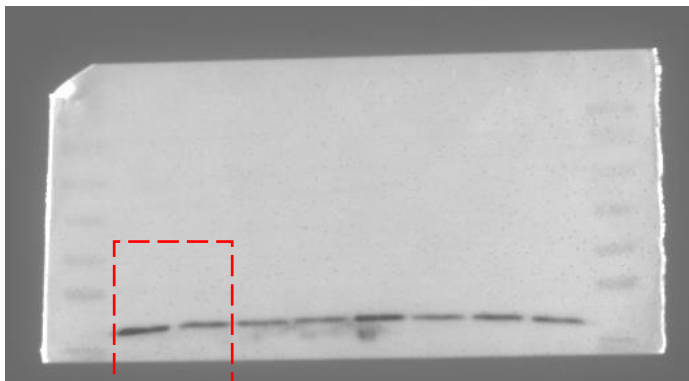

**21kDa**

**$\beta$ -actin**

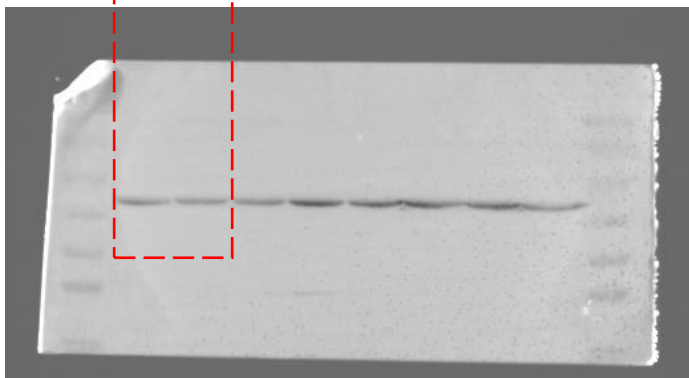

**42kDa**

Supplement: S1 Raw images — (PDF) [file pone.0294227.s002.pdf]
